# Prevalence of Hepatitis B in Canadian First-Time Blood Donors: Association with Social Determinants of Health

**DOI:** 10.3390/v16010117

**Published:** 2024-01-13

**Authors:** Sheila F. O’Brien, Behrouz Ehsani-Moghaddam, Mindy Goldman, Steven J. Drews

**Affiliations:** 1 Epidemiology & Surveillance, Canadian Blood Services, Ottawa, ON K1G 4J5, Canada; behrouz.ehsani-moghaddam@blood.ca; 2 School of Epidemiology & Public Health, University of Ottawa, Ottawa, ON K1G 192, Canada; 3 Centre for Studies in Primary Care, Department of Family Medicine, Queens University, Kingston, ON K7L 3N6, Canada; 4 Donation and Policy Studies, Canadian Blood Services, Ottawa, ON K1G 4J5, Canada; mindy.goldman@blood.ca; 5 Department of Pathology & Laboratory Medicine, Faculty of Medicine, University of Ottawa, Ottawa, ON K1G 192, Canada; 6 Microbiology, Canadian Blood Services, Edmonton, AB T6G 2R8, Canada; steven.drews@blood.ca; 7 Department of Laboratory Medicine & Pathology, Faculty of Medicine & Dentistry, University of Alberta, Edmonton, AB T6G 2E1, Canada

**Keywords:** hepatitis B, blood donors, socioeconomic status, ethnicity, surveillance

## Abstract

Hepatitis B is transmitted sexually, by blood contact, and vertically from mother to child. Chronic hepatitis B is often seen in immigrants from higher-prevalence countries and their Canadian-born children. We assessed the relationship between hepatitis B and social determinants of health. Included were 1,539,869 first-time Canadian blood donors from April 2005 to December 2022. All donations were tested for hepatitis B markers. Logistic regression was fit with chronic hepatitis B as the dependent variable and age, sex, year, and ethnocultural composition and material deprivation quintiles as independent variables. Chronic hepatitis B prevalence was 47.5/100,000 (95% CI 41.5–53.5, years 2017–2022). Chronic hepatitis B prevalence was elevated in males, older age groups, and those living in more materially deprived and higher ethnocultural neighbourhoods. Of 212,518 donors from 2020 to 2022 with race/ethnicity data, chronic hepatitis B prevalence was highest in East Asians. The findings are consistent with infections in immigrants, acquired in their country of origin, in their Canadian-born children and in those with other risks. As blood donors are a low-risk population unaware of their infection and unlikely to seek testing, our results highlight the ongoing public health challenges of diagnosing chronic hepatitis B and treating it when appropriate.

## 1. Introduction

Chronic infection with the hepatitis B virus can lead to cirrhosis and liver cancer [1]. Hepatitis B can be transmitted by blood and other body fluids through injection drug use, blood transfusion, or invasive medical and dental procedures [2]. In adults, hepatitis B is most often a sexually transmitted infection, from which about 90–95% of acute infections resolve spontaneously. In children, infections are usually passed from mother to child, but sometimes by household blood contact. Up to 95% of infants, 50% of children under 5 years, and 10% of adolescents with acute infections will develop chronic hepatitis B [3]. There is no cure for chronic hepatitis B and the prognosis is poorer if acquired in childhood, but it can be treated [4,5].

The World Health Organization set a target of eliminating new hepatitis B infections by 2030 [6]. Worldwide, chronic hepatitis B prevalence decreased by about a third between 1990 and 2019, largely due to neonatal and early-childhood vaccination programs [7]. In Canada, as a high-income, immigrant-receiving country, chronic hepatitis B is most often reported in immigrants from high-prevalence countries and is also associated with lower socioeconomic status, intravenous drug use, and Indigenous peoples [8,9,10]. Although universal infant vaccination was recommended in Canada in 2012, it has only been implemented in some provinces, with others having adolescent universal vaccination, with pregnancy screening and vaccination for high-risk newborns [11,12]. Immigrants with chronic infections are not always identified upon arrival and their Canadian-born children may be at higher risk of early-childhood infections progressing to chronic hepatitis B [8,12].

In Canada, blood donors answer screening questions and are deferred for recent history of hepatitis and hepatitis B risk factors, and all blood donations are tested for hepatitis B. We began collecting self-reported race/ethnicity data from donors in 2020. We have previously reported on data from 2005 to 2020, demonstrating that chronic hepatitis B is low in first-time blood donors (62 per 100,000 donors in 2020), but higher in males and in the vaccine-ineligible birth cohort [12]. The proportion of first-time donors with resolved hepatitis B infections was higher (1450 per 100,000). Blood donors are a population of particular interest since they are unaware of their infection and negative for recent risk factors. We now evaluate the association of chronic and resolved hepatitis B infections with residential neighbourhood ethnocultural composition and material deprivation, and describe the ethnicity of donors with chronic and resolved hepatitis B.

## 2. Materials and Methods

### 2.1. Blood Donor Data

Canadian Blood Services collect blood donations from all provinces in Canada except Quebec. There is no blood collection in the Northern Territories. There are donor clinics in all major cities, as well as many smaller municipalities. Blood is also collected from mobile clinics to reach many smaller towns. Donors must be 17 years of age to donate and there is no upper age limit. Before each donation, the safety of the donor and risk factors for infectious diseases are assessed based on a screening questionnaire and follow-up questions administered by a trained screener. Starting in 2020, all donors were asked, in a voluntary question, to select their race/ethnic group from the following choices: White, South Asian, Asian (East or other), Indigenous, Arab, Black, Latin American, or Other. Donors were deferred permanently for a history of injection drug use or for receiving payment for sex at any time in the past. Men who had sex with men were effectively ineligible if they were in a relationship because all were permanently deferred if they had had a male sexual partner at any time since 1977; in 2013, a temporary deferral period was put in place and was progressively reduced until its removal in 2022. Donors were also deferred temporarily for a history of jaundice, tattoo, skin piercing, acupuncture, transfusion, needlestick injury, previous sexual contact with persons who inject drugs (PWID), paying money or drugs for sex, or recent vaccination for hepatitis B. The study period included all first-time allogeneic donors from April 2005 to 31 December 2022. The Epidemiology Donor Database included each donor’s date of birth, sex, and residential postal code, as well as dates of donation and test results. From 1 January 2020 to 31 December 2022, the donor’s race/ethnic group was also included.

### 2.2. Hepatitis B Testing

All blood donations were tested for hepatitis B surface antigen (HBsAg) using the Abbott PRISM HBsAg immunoassay (Abbott Diagnostics Division, Wiesbaden, Germany). All blood donations were also tested for total antibodies to hepatitis B core antigen (anti-HBc) using the Abbott PRISM HBcore assay (Abbott Diagnostics Division, Wiesbaden, Germany). Starting in 2011, all donations were also tested for hepatitis B virus nucleic acid (HBV NAT) using the Roche Cobas MPX nucleic acid test (Roche Diagnostics International Ltd., Rotkreuz, Switzerland) in pools of six. If a pool was positive, individual samples were tested in singlet. HBsAg-reactive samples were confirmed via neutralization and anti-HBc being unconfirmed. The donors were informed of a positive result by letter and indefinitely deferred. Beginning in 2014, donors who tested positive for HBsAg, but not for HBV NAT or anti-HBc, and reported recent hepatitis B vaccination were excluded from the data.

### 2.3. Chronic and Resolved Hepatitis B

Donors were considered likely to have a chronic hepatitis B infection if they had a positive HBsAg test and a reactive anti-HBc test (with or without a reactive HBV NAT test). We note that acute hepatitis B infection cannot be ruled out with single HBsAg and anti-HBc-reactive results. Donors were considered likely to have a resolved hepatitis B infection if they had a reactive anti-HBc, but not HBsAg or HBV NAT.

### 2.4. Data Management and Statistics

#### Epidemiology Donor Database

The proportions of first-time donors with chronic hepatitis B or resolved hepatitis B were calculated with 95% confidence intervals using the Exact method. The years were grouped into four periods (2005–2008; 2009–2012; 2013–2016; 2017–2022) and four age groups (17–30, 31–40, 41–50, >50). Some provinces were grouped together due to small populations, such that the Atlantic region was Nova Scotia, New Brunswick, Newfoundland, and Prince Edward Island, and the Prairies region was Saskatchewan and Manitoba. Logistic regression models were fit for the full dataset: one with chronic hepatitis B as the dependent variable and one with resolved hepatitis B. For each model, year, sex, age group, region, material deprivation index, and ethnocultural index were dependent variables. Interaction terms were fit. For 2000 to 2022, simple logistic regression models were fit with chronic hepatitis B or resolved hepatitis B as the dependent variable and race/ethnicity as the independent variable.

The Pampalon material deprivation index (MDI) was used to estimate socioeconomic status [13,14]. Material deprivation is associated with an insecure job situation, insufficient income, and low education. The CanMarg ethnocultural composition index estimates the ethnic concentration of the donor’s residential neighbourhood [15,16]. This index is based on the proportion of people who are recent immigrants and the proportion of people who are visible minorities. The MDI and the ethnocultural composition index were derived from the Statistics Canada census in 5-year periods, aggregated from postal codes to the dissemination area level (the smallest geographic unit available in the Canadian census, considering 400–700 persons), and were categorized into quintiles: from least deprived (1) to most deprived (5) (MDI), and from lowest ethnocultural concentration (1) to the highest areas (5) (Ethnocultural Composition Index). All analyses were carried out using SAS software (Version 9.3, SAS Institute, NC, USA).

## 3. Results

**Figure 1:**
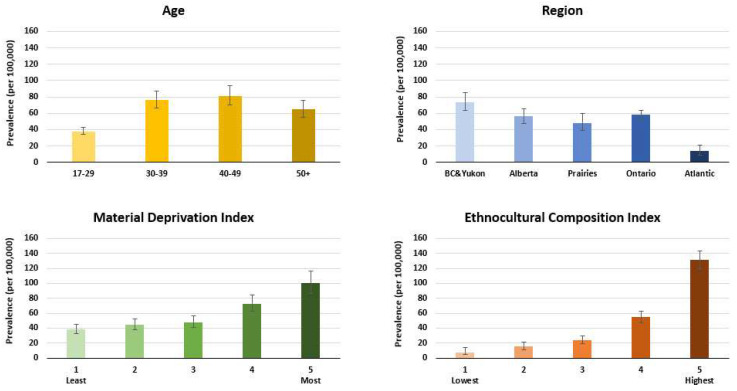
Chronic hepatitis B prevalence by age group, region, material deprivation index, and ethnocultural composition index (2005–2022) (850 chronic HBV cases of 1,539,869 first-time donors).

**Figure 2:**
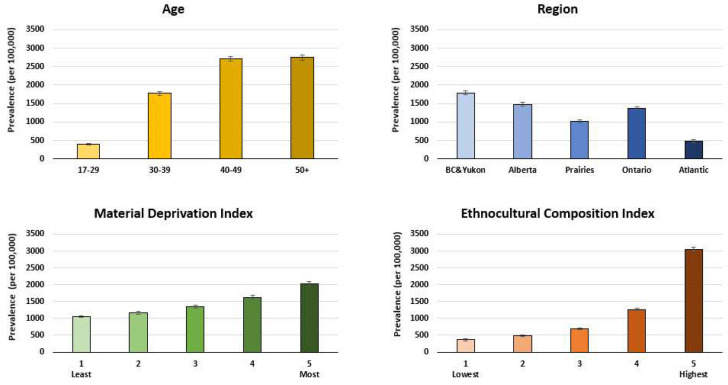
Resolved hepatitis B prevalence by age, region, material deprivation index, and ethnocultural composition index (2005–2022) (20,667 resolved HBV cases of 1,539,869 first-time donors).

There were 1,539,869 first-time blood donors included in the analysis (demographics shown in Table 1). Chronic hepatitis B decreased slightly from 62.2 per 100,000 donors (95% CI 53.6–70.8) in the period 2005 to 2008 to 47.5 (95% CI 41.5–53.5) in the period 2017 to 2022 (*p* < 0.0001). The logistic regression model predicting chronic hepatitis B infection is shown in Table 2. For chronic hepatitis B, the risk ratios indicated that prevalence was higher in males, all age groups over 30 years, in those living in materially deprived neighbourhoods, and those living in neighbourhoods with higher ethnocultural composition (see Table 2 and Figure 1). The prevalence of resolved hepatitis B was about 20–30 times higher than for chronic hepatitis B, with 1240 per 100,000 donors (95% CI 1200–1280) over the period 2005–2008 and increasing to 1470 (95% CI 1430–1500) by the period 2017–2022. The logistic regression model predicting resolved hepatitis B infection is shown in Table 3. For resolved infections, the risk ratios indicated that prevalence was higher in males, in progressively older age groups, and in those living in British Columbia or Alberta compared with Ontario, and was lower in those living in the Atlantic region, among those living in more materially deprived neighbourhoods, and in those living in neighbourhoods with higher ethnocultural composition (see Table 3 and Figure 2). Notably, the risk ratios of being male and living in the highest quintile of ethnocultural composition were each about double in the chronic hepatitis B model compared with the resolved infection model.

**Figure 3:**
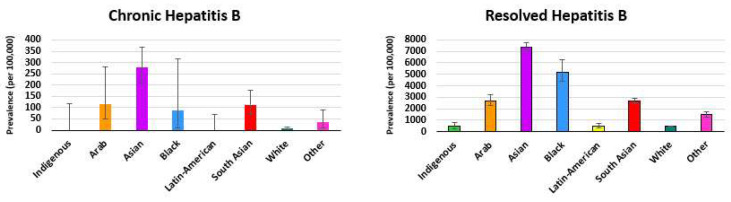
Prevalence of chronic hepatitis B and resolved hepatitis B in donors by self-reported race/ethnicity (2020–2022). Note: The y axis scale is larger for resolved hepatitis B.

There were 212,518 first-time donors over the period from 2020 to 2022 when donors were asked to indicate their race/ethnicity, of whom 184,471 provided their race/ethnicity and were included in the analysis. Chronic hepatitis B prevalence was higher than White donors for all race/ethnicities except Indigenous and Latin American, for which there were no cases. The highest point prevalence was in Asians (East or other) at 277.8 per 100,000, followed by Arabs at 117.0 per 100,000 and South Asians at 113.4 per 100,000, although these were not significantly different from each other (see Figure 3). Resolved hepatitis B prevalence was highest in Asians (East or other) at 7340.5 per 100,000 followed by Black individuals at 5210.2 per 100,000, Arab at 2690.1 per 100,000, and South Asian at 2631.0 per 100,000. Overall, 7.6% of East Asian, 5.3% of Black, 2.8% of Arab, and 2.7% of South Asian individuals had either chronic or resolved hepatitis B and were permanently deferred. The simple logistic regression model outputs are shown in Supplemental Materials, Tables S1 and S2.

## 4. Discussion

Over nearly 17 years of monitoring, we report that there are 47.5 per 100,000 chronic hepatitis B infections and about 30 times more (1470 per 100,000) resolved infections in first-time blood donors. Both chronic and resolved hepatitis B infection prevalences were higher in those living in more materially deprived and in higher ethnocultural composition neighbourhoods. Over the 3 years when self-reported race/ethnicity data were collected, the prevalence of both chronic hepatitis B and resolved infection was highest in East Asians.

Blood donors are at low risk of hepatitis B because they are screened for risk factors prior to donation. The low but fairly steady rate of chronic infections year after year at about 50 per 100,000 first-time blood donors provides a rare window into the low-risk population unaware of their infection who are unlikely to seek testing from a physician and be diagnosed [12].

The prevalence of chronic hepatitis B in Canada is unclear. Chronic hepatitis B is monitored by the mandatory reporting of cases to public health authorities, but this only captures people who have a reason to be tested [17]. Population surveys, such as the National Health and Nutrition Examination Survey in the US and the Canadian Health Measures Survey (CHMS) in Canada, estimate from a random sample, but are too small for regional estimates and generally add together several years of data for a national estimate [18,19]. In Canada, the most recent results available from CHMS are from 2007 to 2011, which estimated that 400/100,000 of participants had chronic hepatitis B, of whom about half were aware that they had hepatitis B, thus having an awareness about eight times higher than donors [18]. There is a need for a more comprehensive surveillance of chronic hepatitis B in Canada that should include a larger sample of the general population, as well as high-risk groups such as immigrants, people of diverse ethnic backgrounds, and those with known risk factors, such as intravenous drug use history. In Canada, data on resolved infections are not reported to public health authorities in any province, although CHMS reported 4200/100,000 anti-HBc-reactive infections from 2007 to 2011, about four times higher than in donors [18]. The epidemiology of resolved infections is somewhat different from chronic infections because most will be the result of post-puberty horizontal transmission, whereas chronic infections most frequently result from vertical or early-childhood transmission.

In our analysis, donors living in neighbourhoods with a higher ethnocultural composition had higher prevalence of both chronic and resolved hepatitis B. Chronic infections in Canada are more common in immigrants from high-prevalence countries [20,21]. In an Ontario analysis of administrative health data, chronic hepatitis B prevalence was 1.34% overall, but 3.42% among all immigrants and 9.37% among immigrants from highly endemic countries [8]. While specific ethnicities of cultural composition quintiles are not available, our donor race/ethnicity data collected from 2020 to 2022 provide insight. For both chronic and resolved infections, the highest prevalence was among East Asians, consistent with the high prevalence in East Asian countries such as China (7.9%), and Canadian immigration patterns and population studies in Ontario and British Columbia [7,8,21,22]. Elevated prevalence was also seen in South Asian and Arab donors, both associated with regions of high prevalence (2.9% for both), although half that of China [7]. It is noteworthy that prevalence varies markedly within countries and that immigrants to Canada may not reflect the national prevalence in their country of origin [23]. Furthermore, Canadian-born donors could not be distinguished from immigrants in our study and our classifications of ethnicity will include people of multiple countries of origin. Our findings bear some similarity to those reported in other countries in that hepatitis B infections are more prevalent in those born in (or ethnicity associated with) higher-prevalence countries. For example, in US blood donors, higher HBV prevalence is seen in Asian and Black donors; in France, primarily those originating from sub-Saharan Africa; in Australia, from Asia; and in England, those from Eastern Europe, Africa, and Asia [24,25,26,27].

In our study, both chronic and resolved hepatitis B infections were associated with living in more materially deprived neighbourhoods independent of ethnocultural composition. An association between lower socioeconomic status and chronic hepatitis B has been noted in studies in British Columbia and Ontario [8,21]. People with lower socioeconomic status tend to have poorer health literacy and, thus, less understanding of risk factors and poorer access to healthcare [28,29,30]. Consistent with HBV reported case prevalence, resolved infection prevalence was higher in the western provinces of British Columbia and Alberta and lower in the Atlantic region after considering ethnocultural composition and material deprivation [17].

In chronic infections, older age groups were about twice as likely as the youngest age group (17–30) to have an infection, but there was no further trend with increasing age groups beyond that. This is consistent with the high likelihood of chronic infection if acquired at birth or in early childhood. Chronic HBV presents at older ages now due to longer life expectancy, under-diagnosis and under-screening, and universal vaccination programs for younger age groups [9]. In resolved infections, the odds ratio increased with each increasing age group consistent with ongoing transmission over the life span, since HBV infection in adults usually resolves. This is also consistent with higher infection rates in those who did not receive a vaccination because they were adults before childhood vaccination was implemented, as has also been observed in Europe, South Africa, and Asia [31,32,33]. We also observed higher prevalence of chronic and resolved infections in males consistent with most of the literature [4,17,21]. While many of the donor anti-HBc-positive results negative for HBsAg and HBV NAT likely represent resolved infections, some may be occult infections. This is clinically important because occult infections can reactivate with immune suppression [34].

Our study has some important limitations. Neighbourhood indicators of material deprivation and ethnocultural composition may not always reflect the attributes of individuals. They each provide five quintiles of increasing concentration of individuals with the attribute based on national census data. For example, the ethnocultural composition index stratifies the Canadian population based on the proportion of people who are immigrants and and/or self-reported ethnic minorities into five quintiles from high proportion to low proportion. A particular donor may or may not share these attributes. An important limitation of blood donor data is that only one sample is available for testing. This means that follow-up testing is not possible and the amount of supplementary testing that can be carried out is also limited. Different study designs would be needed to elucidate biological or immunological mechanisms of infection and to address clinical decision making. We considered donors with HBsAg-positive and anti-HBc-reactive results to have chronic infections, but acute hepatitis B cannot be ruled out with a single-occasion test. However, donors are screened and deferred for many recent risk factors and incident hepatitis B infections are very rare in repeat donations [25,35,36]. Anti-HBc was defined as total immunoglobulin with no confirmatory assay, and anti-HBs data were not available; thus, false positives and acute infections cannot be ruled out [37]. Without repeat blood sampling or history of symptoms, definitive case definitions could not be met [38]. Resolved infections were assumed when there were anti-HBc-reactive results but negative results for HBsAg or HBV NAT. However, some may have had occult infections with low intermittent HBV DNAemia [39]. In addition, as only 3 years of data are currently available regarding the ethnic group of the donors, a longer follow-up will be needed. We note that additional data are required to inform donor policy.

Our recent publication of interviewed donors with chronic hepatitis B suggested that there is a segment of Canadians unaware of their hepatitis B infection, comprising immigrants, their children, and those with other risks [12]. The diversity of risk factors is consistent with the independent association of chronic hepatitis B and ethnocultural and materially deprived neighbourhoods. Canadian guidelines in 2012 and 2018 recommended universal infant or neonatal vaccination and the screening of all pregnant people, immigrants in the pre-immigration health check, immigrants’ unvaccinated Canadian-born children, those with household contacts, and those with high-risk behaviours [2,11]. It is too soon to see the full impact of these guidelines. The screening of immigrants in community settings who were not tested in the immigration process would be moderately cost-effective as an interim measure [40,41]. Of note, imperfect adherence to the guidelines leaves some people at risk. So far, only four provinces have implemented infant screening programs, while others have pre-adolescent and high-risk programs. Vaccination is effective, but the uptake is not perfect [42,43,44]. Pregnancy screening misses some people with chronic hepatitis B, as does pre-immigration screening [8,45,46]. The current thinking is that even with scaled-up intervention strategies, for immigrant-receiving countries such as Canada, the World Health Organization goal of hepatitis B elimination by 2030 is not realistic [47].

## 5. Conclusions

Our analysis shows that, over the past 17 years, there has been low but steady chronic hepatitis B prevalence in first-time blood donors that is associated with material deprivation and ethnocultural concentration. This is consistent with infections in immigrants, acquired in their country of origin, in their children, and in those with other risks. Such infections mostly existed prior to the release of current screening guidelines and/or were missed due to imperfect guideline adherence. As blood donors are a low-risk population unaware of their infection and unlikely to seek testing, our results highlight the ongoing public health challenge in diagnosing chronic hepatitis B and treating it when appropriate.

## Figures and Tables

**Table 1:** Percentage of donors with chronic hepatitis B, resolved hepatitis B, and all donors by sex, age group, and region.

	Chronic Hepatitis B (N = 850)	Resolved Hepatitis B (N = 20,667)	All Donors (N = 1,538,869)
Sex			
Female	25.88	39.86	53.77
Male	74.12	60.14	46.23
Age Group			
17-30	35.88	15.34	52.23
31-40	24.47	23.44	17.70
41-50	21.76	29.90	14.78
>50	17.88	31.32	15.30
Region			
British Columbia	20.82	20.80	15.58
Alberta	18.24	19.83	17.97
Prairies	9.18	7.96	10.53
Ontario	49.53	48.15	46.80
Atlantic	2.24	3.25	9.12

**Table 2:** Output from logistic regression model with chronic hepatitis B as the dependent variable (850 chronic HBV cases of 1,539,869 first-time donors).

Variable	Relative Risk (RR)	95% CI	*p*-Value
Male (compared with female)	2.98	2.53, 3.50	<0.0001
Age (compared with 17–30)			
31–40	1.83	1.52, 2.20	<0.0001
41–50	2.16	1.78, 2.62	<0.0001
>50	2.04	1.66, 2.52	<0.0001
Region (compared with Ontario)			
British Columbia	1.15	0.96, 1.39	0.1323
Alberta	1.07	0.88, 1.31	0.4911
Prairies	1.19	0.93, 1.53	0.1669
Atlantic	0.62	0.38, 1.01	0.0557
Year (compared with 2017–2022)			
2005–2008	1.66	1.37, 2.01	<0.0001
2009–2012	1.30	1.07, 1.58	0.0081
2013–2016	0.98	0.80, 1.20	0.8399
Material Deprivation Quintile (compared with quintile 1)			
2	1.22	0.96, 1.55	0.1103
3	1.18	0.92, 1.52	0.1945
4	1.61	1.26, 2.05	0.0001
5	2.11	1.65, 2.69	<0.0001
Ethnocultural Composition Quintile (compared with quintile 1)			
2	1.67	0.84, 3.33	0.1406
3	2.66	1.39, 5.08	0.0030
4	6.37	3.42, 11.88	<0.0001
5	12.35	6.68, 22.82	<0.0001

**Table 3:** Output from logistic regression model with resolved hepatitis B as the dependent variable (20,667 resolved HBV cases of 1,539,869 first-time donors).

Variable	Relative Risk (RR)	95% CI	*p*-Value
Male (compared with female)	1.46	1.42, 1.51	<0.0001
Age (compared with 17–30)			
31–40	4.09	3.91, 4.28	<0.0001
41–50	6.93	6.64, 7.23	<0.0001
> 50	8.22	7.88, 8.57	<0.0001
Region (compared with Ontario)			
British Columbia	1.07	1.03, 1.11	0.0002
Alberta	1.12	1.08, 1.16	<0.0001
Prairies	0.99	0.94, 1.05	0.8002
Atlantic	0.73	0.68, 0.80	<0.0001
Year (compared with 2017–2022)			
2005–2008	0.85	0.82, 0.88	<0.0001
2009–2012	0.88	0.84, 0.91	<0.0001
2013–2016	0.90	0.87, 0.93	<0.0001
Material Deprivation Quintile (compared with quintile 1)			
2	1.09	1.04, 1.14	<0.0001
3	1.19	1.14, 1.25	<0.0001
4	1.33	1.27, 1.39	<0.0001
5	1.56	1.49, 1.64	<0.0001
Ethnocultural Composition Quintile (compared with quintile 1)			
2	1.28	1.16, 1.42	<0.0001
3	1.86	1.69, 2.05	<0.0001
4	3.50	3.19, 3.85	<0.0001
5	7.83	7.15, 8.58	<0.0001

## Data Availability

Summary data are available upon request from sheila.obrien@blood.ca.

## Supplementary Materials

The following supporting information can be downloaded at: https://www.mdpi.com/article/10.3390/v16010117/s1, Table S1. Output from simple logistic regression model with chronic hepatitis B as the dependent variable and race/ethnicity as the independent variable. Table S2. Output from simple logistic regression model with resolved hepatitis B as the dependent variable and race/ethnicity as the independent variable.
