# Supplementary material for: Prevalence of Hepatitis B in Canadian First-Time Blood Donors: Association with Social Determinants of Health
Source: Viruses. 2024 Jan 13;16(1):117. doi: 10.3390/v16010117 (PMC11326446; doi:10.3390/v16010117)
Supplement: Supplementary file 1 [file viruses-16-00117-s001.zip › viruses-2788858-supplementary.pdf]

Table S1. Output from simple logistic regression model with chronic hepatitis B as the dependent variable and race/ethnicity as the independent variable.

Variable	Relative Risk	95% CI	P Value
Aboriginal	_		
Arab	12.72	3.92, 41.30	<0.0001
Asian (East and other)	35.52	17.41, 72.46	<0.0001
Black	11.91	2.57, 55.08	0.0015
Latin American	-		
South Asian	15.42	7.02, 33.86	<0.0001
Other	4.74	1.46, 15.38	0.0096

Note: White is the comparator

Table S2. Output from simple logistic regression model with resolved hepatitis B as the dependent variable and race/ethnicity as the independent variable.

Variable	Relative Risk	95% CI	P Value
Aboriginal	1.15	0.68, 1.96	0.5947
Arab	6.51	5.34, 7.93	<0.0001
Asian (East and other)	18.95	17.19, 20.88	<0.0001
Black	13.66	11.31, 16.50	<0.0001
Latin American	1.28	0.86, 1.90	0.2297
South Asian	6.70	6.17, 7.93	<0.0001
Other	1.43	2.99, 4.19	<0.0001

Note: White is the comparator
